# Gonadal Function in Boys with Bilateral Undescended Testes

**DOI:** 10.1210/jendso/bvad153

**Published:** 2023-12-12

**Authors:** Angela K Lucas-Herald, Khalid I Alkanhal, Emma Caney, Iman Malik, Malika Alimussina, Jane D McNeilly, Timothy Bradnock, Boma Lee, Mairi Steven, Martyn Flett, Stuart O’Toole, Ruth McGowan, S Faisal Ahmed

**Affiliations:** Developmental Endocrinology Research Group, University of Glasgow, Royal Hospital for Children, Glasgow G51 4TF, UK; Developmental Endocrinology Research Group, University of Glasgow, Royal Hospital for Children, Glasgow G51 4TF, UK; Obesity and Endocrine Metabolism Center, King Fahad Medical City, 58046 Riyady 11525, Saudi Arabia; Developmental Endocrinology Research Group, University of Glasgow, Royal Hospital for Children, Glasgow G51 4TF, UK; Developmental Endocrinology Research Group, University of Glasgow, Royal Hospital for Children, Glasgow G51 4TF, UK; Developmental Endocrinology Research Group, University of Glasgow, Royal Hospital for Children, Glasgow G51 4TF, UK; Department of Clinical Biochemistry, Queen Elizabeth University Hospital, Glasgow G51 4TF, UK; Department of General Paediatric Surgery, Royal Hospital for Children, Glasgow G51 4TF, UK; Department of Paediatric Urology, Royal Hospital for Children, Glasgow G51 4TF, UK; Department of Paediatric Urology, Royal Hospital for Children, Glasgow G51 4TF, UK; Department of Paediatric Urology, Royal Hospital for Children, Glasgow G51 4TF, UK; Department of Paediatric Urology, Royal Hospital for Children, Glasgow G51 4TF, UK; Developmental Endocrinology Research Group, University of Glasgow, Royal Hospital for Children, Glasgow G51 4TF, UK; West of Scotland Centre for Genomic Medicine, Queen Elizabeth University Hospital, Glasgow G51 4TF, UK; Developmental Endocrinology Research Group, University of Glasgow, Royal Hospital for Children, Glasgow G51 4TF, UK

**Keywords:** cryptorchidism, orchidopexy, hCG, gonadal function

## Abstract

**Background:**

Bilateral undescended testes (BUDT) may be a marker of an underlying condition that affects sex development or maturation.

**Aims:**

To describe the extent of gonadal dysfunction in cases of BUDT who had systematic endocrine and genetic evaluation at a single tertiary pediatric center.

**Methods:**

A retrospective review was conducted of all boys with BUDT who had endocrine evaluation between 2008 and 2021 at the Royal Hospital for Children, Glasgow (RHCG). Continuous variables were analyzed using Mann–Whitney U and non-continuous variables using Fisher’s exact, via Graphpad Prism v 8.0. Multivariable logistic regression was used to identify any associations between groups. A *P* < .05 was considered statistically significant.

**Results:**

A total of 243 bilateral orchidopexies were performed at RHCG between 2008 and 2021. Of these 130 (53%) boys were seen by the endocrine team. The median (range) age at first orchidopexy was 1 year (0.2, 18.0) with 16 (12%) requiring re-do orchidopexy. The median External Masculinization Score of the group was 10 (2, 11) with 33 (25%) having additional genital features. Of the 130 boys, 71 (55%) had extragenital anomalies. Of the 70 who were tested, a genetic abnormality was detected in 38 (54%), most commonly a chromosomal variant in 16 (40%). Of the 100 who were tested, endocrine dysfunction was identified in 38 (38%).

**Conclusion:**

Genetic findings and evidence of gonadal dysfunction are common in boys who are investigated secondary to presentation with BUDT. Endocrine and genetic evaluation should be part of routine clinical management of all cases of BUDT.

Undescended testis is a common congenital anomaly with a birth prevalence of between 1.5% and 8% of newborns [1]. The majority will have a unilateral undescended testis, and only 1/10 of these newborns will present with bilateral undescended testes (BUDT) [2]. Undescended testes, especially when they are bilateral or when they are associated with other genital anomalies such as micropenis or hypospadias, may be a marker for a genetic condition that affects sex development such as hypogonadotrophic hypogonadism [3] or a disorder of gonadal development, androgen synthesis, or androgen action [4]. In addition to being a marker of an underlying condition, undescended testes themselves may have an impact on long-term gonadal outcome including pubertal development [5], fertility [6], and tumor development [7]. While recent guidance recommends that boys with BUDT should undergo expert evaluation [8], and there is evidence that boys with undescended testes may display some evidence of gonadal dysfunction [9-11], there is little consensus on the extent of the evaluation required in these cases.

The aim of the current study was to describe the spectrum of abnormalities encountered in boys undergoing comprehensive endocrine evaluation at a single tertiary pediatric center to provide a deeper insight into the prevalence of endocrine abnormalities in these cases and consider the rationale for investigating this group of boys.

## Patients and Methods

A retrospective review was conducted on the clinical records of all boys with BUDT who had undergone evaluation over a 14-year period from January 1, 2008, to December 31, 2021, by the endocrine service at the Royal Hospital for Children, Glasgow. Boys were selected by searching the hospital's operating theater database, which lists all procedures undertaken for all orchidopexies undertaken within the desired time period. An undescended testis was defined by its presence out with the scrotum, and the location of the testis was determined based on its site after induction of anesthesia. For the purposes of analysis, testes were classified as abdominal if either testis was identified in the abdomen, regardless of an inguinal position of the contralateral testis. Boys with unilateral undescended testis, retractile testes, or anorchia were excluded from the study. The external genitalia were described using the External Masculinization Score (EMS) [12]. Data collection was undertaken in accordance with the ethics guidance of the National Health Service Health Research Authority decision tool (https://www.hra-decisiontools.org.uk/research/) as an evaluation of the current clinical service provision.

### Stimulation Tests and Assays

The protocol for human chorionic gonadotrophin (hCG) stimulation included hCG at a dose of 1500 U IM on days 1, 2, and 3 in the first week of the test. A blood sample for testosterone (T) was collected on days 1 and 4 of the test. In some cases, a prolonged hCG stimulation test was conducted, where further injections were administered twice a week for 2 further weeks following which a blood sample was collected on day 22 [13]. A normal T response to hCG stimulation test was defined as per previously published studies where adequate T response would be greater than 3.5 nmol/L at day 4 of the test and greater than 9.5 nmol/L on day 22 [13].

For samples from 2008 to 2013, serum T was measured using a chemiluminescent microparticle immunoassay on the Abbott Architect analyser (RRID AB_2848165, Abbott Laboratories Diagnostics, Santa Clara, CA, USA) after solvent extraction. Functional sensitivity was 0.5 nmol/L and the inter- and intra-assay coefficients of variation (CVs) were <8%. For samples after 2013, T was measured using liquid chromatography with tandem mass spectrometry via the Xevo TQS Tandem Mass Spectrometer (Waters Corporation, Milford, MA, USA) with a functional sensitivity of 0.1 nmol/L. Steroids were extracted from serum/plasma using Biotage supported liquid extraction, automated on the CTC PAL (MicroLiter Analytical Supplies Inc, Suwanne, GA, USA), followed by ultra-performance liquid chromatographic separation. The inter- and intra-assay CVs were also <8%.

For samples between 2008 and 2010, anti-Müllerian hormone (AMH) was measured using an enzymatically amplified 2-site immunoassay (Active MIS/AMH Elisa DSL-10_14400, Diagnostics Systems Laboratories, Webster, TX, USA), with a functional sensitivity of 4 pmol/L and intra- and inter- assay CVs of 5% and 8%, respectively [14]. For samples between 2010 and 2016, AMH was measured using a semi-automated Beckman Gen II ELISA assay (RRID AB_2923005, Beckman Coulter, Indianopolis, IN, USA) with a functional sensitivity of 4 pmol/L and inter- and intra-assay CVs of <5%. For samples after 2016, AMH was measured using enzyme-linked immunosorbent assay using the fully automated Beckman Access MDL assay (RRID AB_2892998, Beckman Coulter), with a functional sensitivity of 1 pmol/L and inter- and intra-assay CVs of <5%. Age-related AMH reference ranges were used to create centiles, and a low AMH was defined as below the 5th centile for age [13, 14].

The LH releasing hormone (LHRH) stimulation test included collection of blood for LH and FSH followed by administration of LHRH 100 micrograms IV and additional collection of blood for LH and FSH at 30 and 60 minutes [15]. Hypogonadotrophic hypogonadism was defined as a lack of response to LHRH stimulation (no increase from basal level). Gonadal failure was suspected in those cases where basal LH and FSH were high or their responses to LHRH were elevated (>10mU/L). LH and FSH were measured on the Abbott Architect ci1600 using chemiluminescent microparticle immunoassays (RRIDs AB_2813909 and AB_2813910, Abbott Laboratories Diagnostics, Santa Clara, CA, USA), with a functional sensitivity of 0.1 IU/L and inter- and intra-assay CVs of <5% for both.

### Genetic Analysis

DNA extraction from peripheral blood samples was performed as per clinical genetic testing standards for patients undergoing routine testing. Single nucleotide polymorphism microarray was performed using Illumina CytoCNP 850k v1.2 BeadChip. Data were analysed with BlueFuse Multi v4.4, BeadArray v2 algorithm. Analysis parameters were set to detect copy number variations ≥ 10 kb within OMIM morbid/Developmental Disorders Genotype-Phenotype Database genes, losses ≥ 200 kb or gains ≥ 500 kb. Any abnormal findings were compared with data held in the DECIPHER database (https://decipher.sanger.ac.uk/). Where appropriate, Differences of Sex Development (DSD) panel testing was performed using a dedicated 56-gene panel as previously described [13].

### Statistical Analysis

The data were analysed using PRISM, version 8.1.2. Continuous variables were analyzed using Mann–Whitney U and noncontinuous variables using Fisher’s exact. The Kruskal–Wallis test was used when more than 2 groups were compared. Multiple linear regression was used to identify associations between groups. A *P* < .05 was considered statistically significant.

## Results

### Characteristics of the Study Population

A total of 243 bilateral orchidopexies were performed at RHCG between 2008 and 2021. Of these, 130 (53%) boys were seen by the endocrine team (Table 1). There was no statistically significant difference in those who were referred to the endocrine team compared to those who were not in terms of EMS (*P* = .07); however, those who were referred were more likely to have additional genital features (25% vs 5%, *P* < .0001) and presence of extragenital features (55% vs 9%, *P* < .0001). Those who were referred to endocrinology also had a greater re-do rate (12% vs 5%).

**Table 1.** Clinical characteristics and testing undertaken

	Total cohort n = 130	Biochemical testing undertaken n = 99 (76% total)		Genetic testing undertaken n = 70 (54% total)	
		Abnormality detected n = 38 (38%)	No abnormality detected n = 61 (62%)	Variant Detected n = 38 (54%)	No variant Detected n = 32 (46%)
Median age (yrs) at orchidopexy (range)	1 (0.2, 18.0)	2 (0.2, 18.0)	1 (1, 8)	1 (1, 10.0)	1 (0.2, 18.0**)**
Median EMS (range)	10 (2, 11)	10 (2, 11)	9 (5, 11)	10 (2, 11)	10 (4, 11)
Location of testes					
Abdominal	23 (18)	10 (26)	14 (23)	9 (23)	8 (25)
Inguinal	99 (76)	23 (60)	45 (73)	27 (71)	21 (65)
1 abdominal, 1 inguinal	8 (6)	5 (14)	2 (4)	2 (6)	3 (10)
Presence of extragenital comorbidities	71 (55)	26 (68)	35 (57)	30 (78)	28 (88)

Abbreviations: EMS, External Masculinization Score.

Of the 130 who were seen by endocrinology, 99 (76%) had bilateral inguinal testes, 23 (18%) had bilateral impalpable testes with the testes located in the abdomen, and 8 (6%) had a combination of an impalpable abdominal testis and an inguinal testis. All 130 cases had bilateral orchidopexy with a median (range) age at first orchidopexy of 1 year (0.2, 18.0). A total of 16 (12%) required re-do orchidopexy, and 6 (5%) of the boys progressed to orchidectomy due to atrophied testes. The median EMS of the group was 10 (2, 11), and 71 (55%) had extragenital anomalies, most commonly neurodevelopmental delay (n = 10, 14%). There was a family history of undescended testes in 14 (11%). In total, 33 (25%) boys had “complex” BUDT with additional genital features. Of these, 21 (16%) boys had coexisting hypospadias [8 (38%) had distal hypospadias and 13 (62%) had proximal hypospadias] and 18 (14%) boys had associated micropenis. Overall, 67 (52%) had ultrasound imaging prior to initial orchidopexy, while only 2 (1%) had magnetic resonance imaging.

### Genetic Evaluation

Genetic analysis was performed in 70 (54%) of the boys with 42 (60%) undergoing the local targeted genetic panel for DSD and 28 (43%) undergoing single nucleotide polymorphism microarray. Of the 70, a genetic alteration was detected in 38 (54%), and, of these, 14 (37%) had complex BUDT. In 16 (40%), this was manifested as a chromosomal variant. These variants are listed in Table 2. Of the further 22 with a genetic alteration identified on genetic testing, 10 (14%) had a *SRD5A2* variant, 8 (12%) had a variant in a gene consistent with hypogonadotrophic hypogonadism [most commonly *CHD7* in 4 (6%)], 2 (3%) had an *AMHR* variant, 1 (1%) had an *AR* variant, and 1 (1%) had a *POLD1* variant. Details of these variants are listed in Table 3. All of the boys who had genetic testing also had some form of endocrine evaluation.

**Table 2.** Chromosomal rearrangements detected in 16 boys within the cohort

Chr	Rearrangement	Position of testes	Additional genital anomalies	Extragenital anomalies or syndromes	Previous reports
2	Deletion 2q14.1	I, I	No	No	Almuzzaini et al, 2020
2	Deletion 2p22.3	I, I	No	Pulmonary valve stenosis, developmental delay	Rocca et al, 2013
3	Deletion 3q14.3	I, I	No	No	Dittner-Moormann et al, 2020
5	Deletion 5p15.2	I, I	No	No	Cerruti Mainardi, 2006
7	Deletion 7q34	I, I	No	Retinal coloboma, developmental delay	Nixon et al, 2017
7	Deletion 7p21.1	I, I	No	No	Fryssira et al, 2011
8	Deletion 8q21.32	I, I	PH, M	Panhypopituitarism	Palomares et al, 2011
11	Deletion 11p11.2	I, I	No	Bilateral talipes	Trajkova et al, 2020
12	Deletion 12q13.12	I, I	No	Hydronephrosis	Nixon et al, 2017
12	Deletion 12q13.3	A, A	No	Cleft lip and palate	Hu et al, 2017
15	Deletion 15q26.2	I, I	No	Prader-Willi syndrome	Bakker et al, 2015
16	Deletion 16p11.2	A, A	No	Arthrogryposis	Chung et al, 2021
20	Deletion 20p.13	A, A	No	No	Nixon et al, 2017
22	Deletion 22q11.2	I, I	No	No	Van Batavia et al, 2019
4 and 10	Unbalanced translocation	I, I	PH	No	Fan et al, 2023
21	Trisomy	A, I	No	Down's Syndrome	Chew and Hutson, 2004

Abbreviations: A, abdominal; chr, chromosome; I, inguinal; M, micropenis; PH, proximal hypospadias.

**Table 3.** Details of genetic variants identified in 22 of the boys

Gene	c.	p.	Classification
AMHR	c.497T > A c.553C > G	p.(Leu166Gln) p.(Gln185Glu)	3
AMHR	c.85 °C > A	p.(Pro284Thr)	3
ANOS1	c.727-2A > G	p.(?)	5
ANOS1	c.1952G > C	p.(Arg651Pro)	3
AR	c.2095G > A	p.(Ala699Thr)	3
CHD7	c.6193C > T	p.(Arg2065Cys)	4
CHD7	c.524C > T	p.(Ser175Leu)	3
CHD7	c.6304G > T	p.(Val2102Phe)	3
CHD7	c.7945G > A	p.(Val2649Ile)	3
FGFR1	c.1569_1572del	p.(Lys523Asnfs*3)	5
FGFR1	2081delT	Phe694Serfs*20	5
NR5A1	c.1379A > T	p.(Gln460Leu)	3
POLD1	Not available	Not available	Not available
SRD5A2	c.698 + 1 > G	p.(?)	3
SRD5A2	c.548-2A > C	p.(?)	4
SRD5A2	c.755 + 1G > T	p.(?)	3
SRD5A2	c.680G > A	p.Gln6	5
SRD5A2	Not available	Not available	Not available
SRD5A2	c.737G > A	p.Arg246Gln	5
SRD5A2	c.698 + 1 > G > T and c.737G > A	p.Arg246Gln	4
SRD5A2	c.154G > A	p.Ala52Thr	4
SRD5A2	Not available	Not available	Not available
SRD5A2	c.698 + 1 > G > T and c.737G > A	p.Arg246Gln	4

### Endocrine Evaluation

Of the 130, 99 (76%) had endocrine biochemistry performed, with a median current age of those who had testing of 6 (4, 8) years compared to 8 (2, 20) years in those with no biochemistry performed, giving a median follow-up time of 2 (1, 18) years. Of those who had testing, the median EMS was 9 (5, 11) compared to 11 (6, 11) in those with no endocrine evaluation. In total, 87 (88%) boys had AMH measured, 82 (83%) had an hCG stimulation test, and 60 (61%) had an LHRH stimulation test. The median (range) age at endocrine evaluation was 1 year (0.1, 17.0). In 61 of the 99 cases (62%), endocrine biochemistry was performed after initial orchidopexy with a range from 1 month prior to surgery to 10.5 years after initial surgery. In comparison to those who had no endocrine biochemistry performed, EMS predicted the likelihood of performing any biochemical tests, whereas original position of testes (*P* = .06), presence of additional comorbidities (*P* = .9), and family history (*P* = .1) did not show any association.

**Figure 1.**
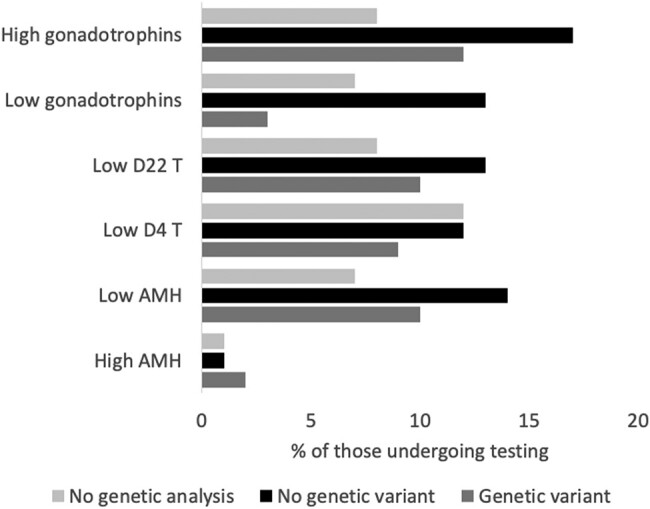
Numbers of boys with abnormal endocrine biochemistry and the presence or absence of any genetic alterations. Abbreviations: AMH, anti-Müllerian hormone; T, testosterone.

Overall, some evidence of endocrine dysfunction was identified in 38 (38%) of the boys who had endocrine testing (Fig. 1). Of these, 6 (16%) had complex BUDT. In the 87 boys who had an AMH performed, this was within the normal range in 56 (64%) at median (range) 574 pmol/L (158, 1614); in 4 (5%) the level was above the normal range at median (range) 1778 pmol/L (962, 2477) and low in 27 (31%) at a median of 41 pmol/L (range <4, 262). Basal gonadotrophins were performed in 86 (66%), and LHRH testing was performed in 60 (46%). FSH was raised in 19 (22%) with a median (range) of 22.6 U/L (11.9, 109.2), and LH was raised in 35 (41%) at a median (range) of 19.7 U/L (10.4, 95.1). A total of 82 boys had an hCG stimulation test, with 52 (63%) of these boys undertaking a prolonged test. Twenty (24%) of the boys had inadequate T response on D4 of the hCG stimulation test with a median (range) D4 T of 0.5 nmol/L (<0.5, 2.5). Sixteen boys (31%) had inadequate T response on D22 of the hCG stimulation with a median D22 T of 1.6 nmol/L (<0.5, 9.2), half of whom had an adequate T response on D4 of the test. Basal T was within the normal range in 3/7 (43%) of the boys receiving first assessment at pubertal age.

**Figure 2.**
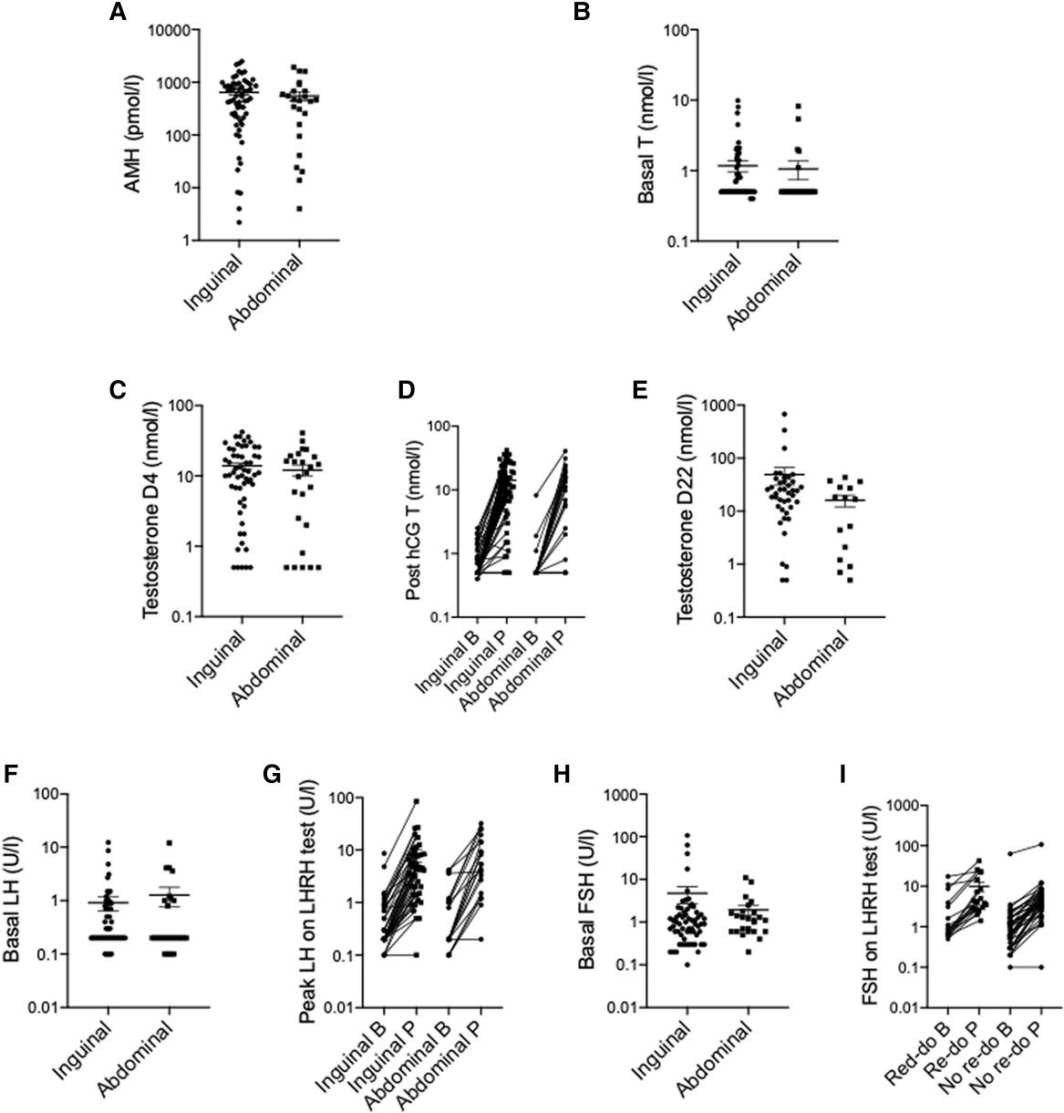
Endocrine findings in boys presenting with bilateral inguinal (n = 99) vs bilateral abdominally situated testes (n = 23). Analysis done via Mann–Whitney U. Abbreviations: B, basal; P, peak.

Of the 99 boys who had endocrine biochemistry performed, there were no differences in AMH, LH, FSH, or T levels between boys with isolated BUDT and those with complex BUDT (Fig. 2). Boys with complex BUDT were more likely to have a genetic variant identified (41% vs 18%, *P* = .01).

**Figure 3.**
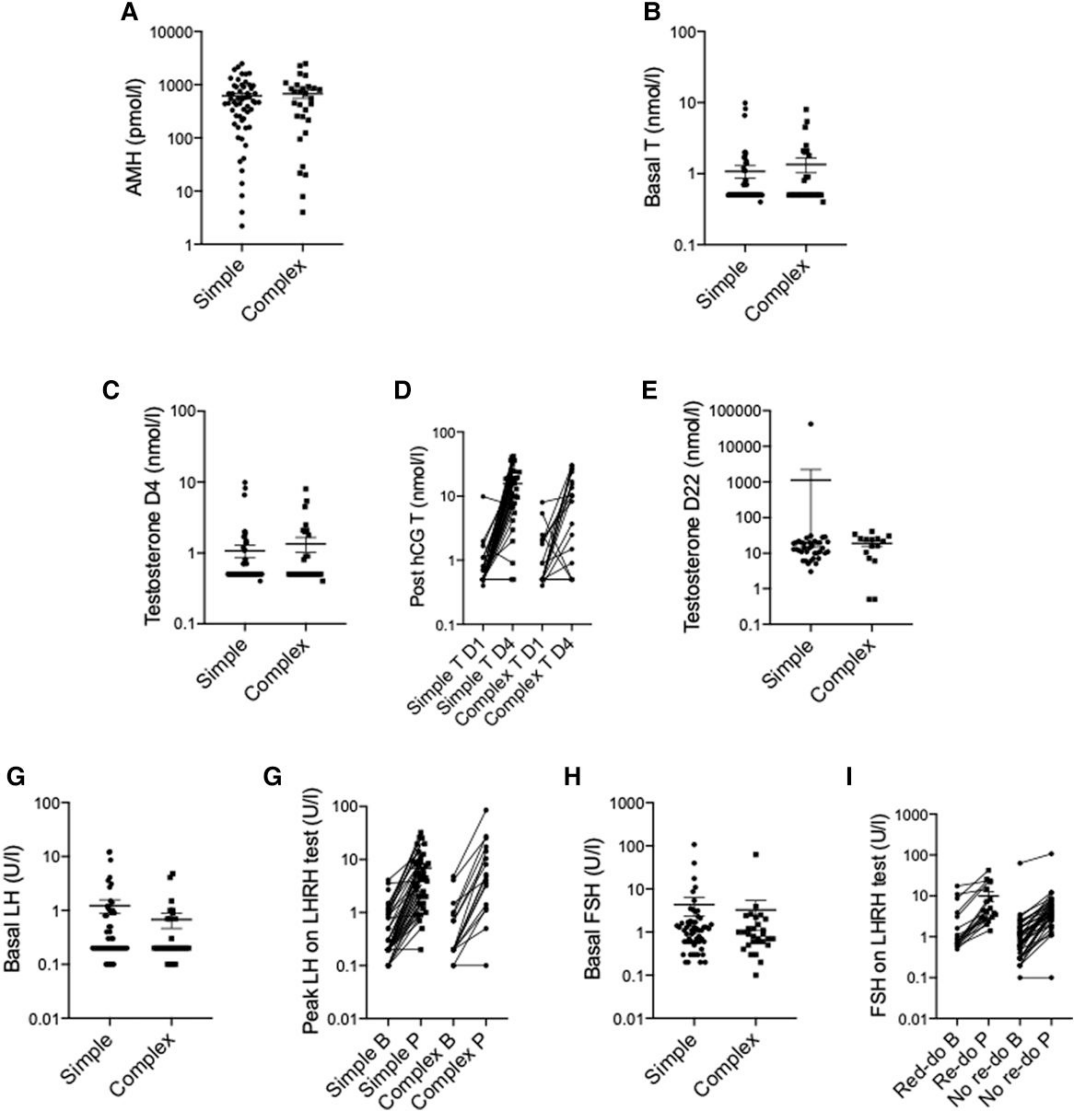
Biochemistry in boys requiring a re-do operation (n = 16) compared to those who did not (n = 114). Analysis done via Mann–Whitney U. Abbreviations: B, basal; P, peak.

**Figure 4.**
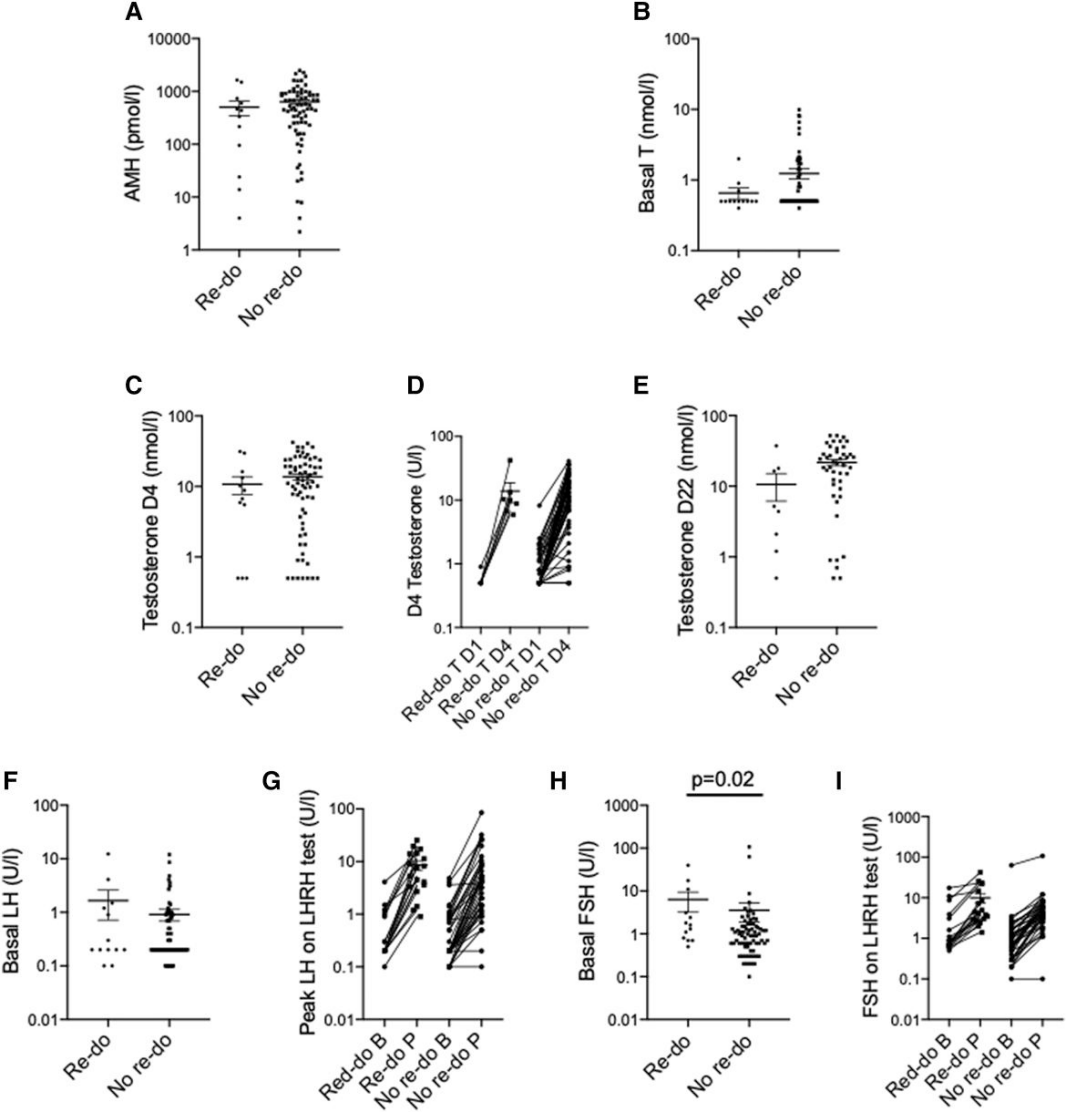
Biochemistry in boys with isolated BUDT (n = 83) compared to those with complex BUDT (n = 16) with additional genital anomalies. Analysis done via Mann–Whitney U. Abbreviations: B, basal; BUDT, bilateral undescended testes; P, peak.

A total of 53 (54%) had complete endocrine evaluation of gonadal function including all 3 tests (AMH, LHRH stimulation, and hCG stimulation). Overall, evidence of gonadal dysfunction was identified in 23 (43%) boys. Of these, the most common abnormality identified was a low AMH in 26 (20%). There were no differences between those with normal and abnormal gonadal dysfunction according to position of testes (*P* = .9) (Fig 3), presence of additional comorbidities (*P* = .09), family history (*P* = .9), or EMS (*P* = .9). In addition, there were no differences in AMH, T, LH, or number of endocrinopathies in those who required a re-do operation compared to those who did not, although basal FSH was higher in those who did require a re-do (1.6 U/L vs 0.9 U/L, *P* = .02) (Fig. 4) and a genetic variant was not more likely to predict the need for a re-do operation. Of the 47 (48%) boys who had some repeat biochemistry taken, none demonstrated any improvement in gonadal function.

### Long-term Outcomes

At the time of the study, of the 130 boys who had been seen by endocrinology, 51 (39%) were over the age of 12 years with a median age of 14.8 yrs (12.0, 24.4). Of these 51, 7 (14%) had required therapy. There were no reported cases of testicular germ cell tumors, and only 3 boys have had sperm analysis undertaken, with this demonstrating azoospermia in 2 (66%). Both of these boys had abdominal testes and evidence of gonadal dysfunction on biochemistry.

## Discussion

This study considers the evaluation of boys with BUDT from a large tertiary pediatric center within the United Kingdom over a 13-year period. We demonstrate variation in the extent to which these children are investigated even within a single center, without any clear clinical predictors regarding this variation. This highlights the importance of a systematic approach to the investigation of such boys, given the high rates of coexisting genetic variants (57%), extragenital abnormalities (55%), and endocrine biochemical dysfunction (38%). Although EMS seemed to be influential in the decision to undertake endocrine biochemistry, there was no association between EMS and likelihood of abnormal biochemistry, which is consistent with findings in a broader range of DSD conditions within the same center [16]. Indeed, given that no clear predictors for the existence of gonadal dysfunction could be identified suggests that, first, further research regarding the mechanisms of gonadal dysfunction in this group is required and, second, guidelines regarding the management of boys born with BUDT should recommend testing for all such individuals. In particular, the presence of extragenital features cannot be relied upon to determine the need for additional testing, as the majority of those with endocrine dysfunction did not have any additional genital features on examination.

In those boys in whom genetic testing via targeted DSD gene panel or chromosomal microarray was undertaken, over half had a genetic variant identified. All of the chromosomal variants identified have previously been reported to be associated with undescended testes [4, 17-29]. It is possible that detection rates may vary also depending on the genetic testing modality employed. This is without the addition of whole exome analysis, which has been shown to increase diagnostic yield for genetic variants to 64% of males with XY DSD [30]. As such, boys with BUDT should be offered chromosomal analysis as well as more detailed molecular genetic testing, particularly in those with complex BUDT, where a variant was most likely to be identified.

Our data demonstrate that one-third of boys with BUDT have inadequate T in response to hCG stimulation tests. Given that half of the boys with low T on prolonged hCG testing had a normal D4 testosterone response, the endocrine evaluation of boys with BUDT should include prolonged hCG testing in preference to standard short hCG stimulation tests. Our findings support previous reports demonstrating that while a short hCG stimulation regimen may exclude some conditions such as 17β-hydroxysteroid dehydrogenase-3 deficiency, some boys with BUDT require prolonged stimulation to thoroughly assess the ability of the testes to produce androgens [31]. In addition, while some studies have demonstrated variable success rates in terms of using hCG to promote testicular descent, recurrence is high using this method, likely due to coexisting anatomical abnormalities in boys with BUDT, most commonly due to abnormal attachment of the gubernaculum [32]. However, hCG testing does provide invaluable information regarding Leydig cell function, which can be used to construct a management plan and counsel boys and their parents regarding likely long-term outcomes. That said, where children are referred and seen around the ages of 3 to 6 months when minipuberty may be occurring, basal T and gonadotrophin levels may prove useful in understanding the function of the testes at that early stage of life. While it is possible that normal results during minipuberty may exclude hypogonadotrophic hypogonadism, it is unclear whether they would obviate the need for further endocrine monitoring for incipient primary gonadal insufficiency.

**Figure 5.**
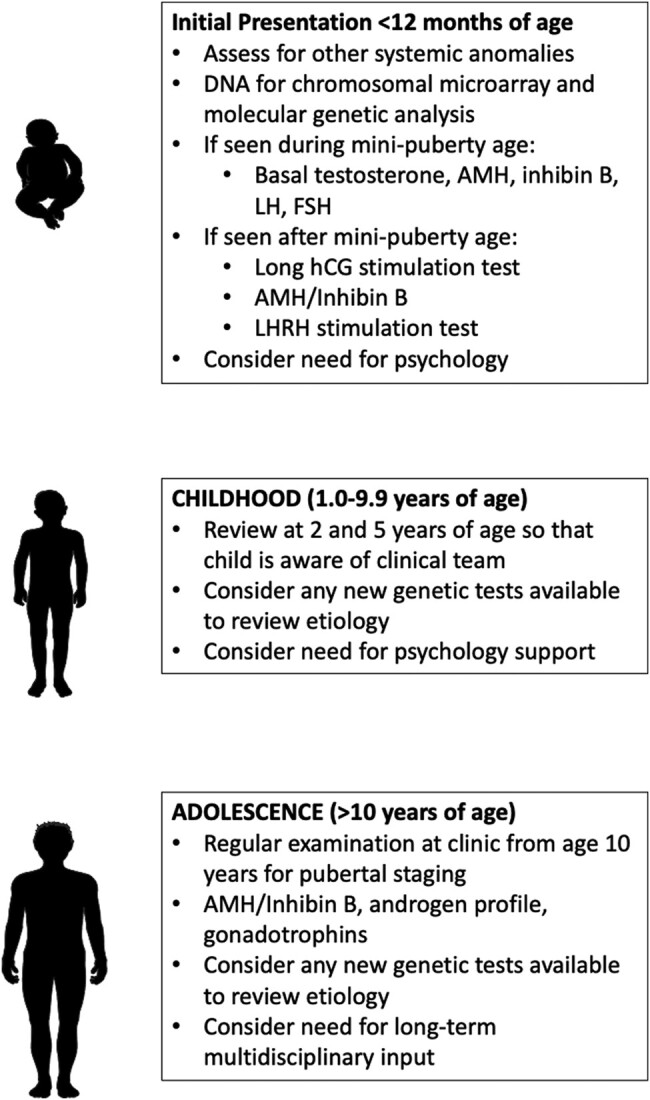
Recommendations for management of boys with bilateral undescended testes.

While this study clearly provides meaningful data on a large number of children with BUDT from a single tertiary center, the retrospective nature of the study means that all affected cases did not undergo a detailed endocrine and genetic evaluation, likely due to different clinical care providers throughout the study. As such, there is a need for examining a larger cohort of cases and for determining if any particular factors raise particular clinical suspicion and alter the decision to refer to endocrinology or to do genetics. It is also possible that the timing of the endocrine evaluation in relation to the age of the child or the orchidopexy may bear a relationship to the results, and this will also require an analysis of a larger number of cases, ideally via a prospective study. The high prevalence of endocrine and genetic findings also highlights the need for ongoing surveillance of long-term outcomes including gonadal function, sex hormone supplementation, fertility, and tumor development through a multidisciplinary team [33]. Of the children who had reached pubertal age at the time of the study, 10% required T supplementation for induction of puberty. It is possible, however, that testicular insufficiency may develop later in life, and, as such, boys with a past history of BUDT should be made aware of the clinical features of hypogonadism in adulthood. The findings in the current study are sufficient for us to recommend that all boys with BUDT follow a standardized pathway for evaluation (Fig. 5) and that this should be similar to what has previously been proposed for differences and disorders of sex development [34]. However, further research is required to evaluate the clinical utility and the long-term benefit to the growing child and adolescent.

In conclusion, gonadal dysfunction is identified in over a third of boys with BUDT. Given there were no clear predictors for development of gonadal dysfunction, we recommend biochemical and genetic analysis for all boys with BUDT.

## Data Availability

Some or all datasets generated during and/or analyzed during the current study are not publicly available but are available from the corresponding author on reasonable request.
